# Narrative elaboration makes misinformation and corrective information regarding COVID-19 more believable

**DOI:** 10.1186/s13104-022-06134-9

**Published:** 2022-06-28

**Authors:** Joanna Greer, Kaitlyn Fitzgerald, Santosh Vijaykumar

**Affiliations:** Department of Psychology, Northumbria University, Newcastle upon Tyne, UK

**Keywords:** Misinformation, COVID-19, Narrative elaboration, Younger adults, Older adults

## Abstract

**Objective:**

People gather information about health topics from online channels oftentimes awash with misinformation. Investigating this problem during the COVID-19 pandemic is important, as the misinformation effect occurs when misleading details are embedded in narratives and questions. This pilot study investigated whether narrative elaboration increases believability in misinformation statements about COVID-19, and willingness to share these statements online.

**Results:**

Results from our online survey (n = 80) demonstrated that narrative elaboration increased believability in both misinformation and accurate statements, with a more pronounced effect on younger adults. Future research may investigate cognitive vulnerabilities imposed by elaborate narratives embedded in online health misinformation with increased attention on developing misinformation resilience among younger adults.

**Supplementary Information:**

The online version contains supplementary material available at 10.1186/s13104-022-06134-9.

## Introduction

Misinformation is broadly understood an umbrella term to include all false or inaccurate information that is spread in social media [1]. Since the outbreak of the SARS-CoV-2 virus in 2020 online platforms have been awash with COVID-19 pandemic-related information, however these are often abound with misinformation containing content which is misleading or false [2] (see [3] for an evaluation of online source credibility). Thus, the influence of misinformation is highly impactive as differences in believability between credible content and misinformation can cause a split between scientific consensus and public opinion [4]. Refutation text, such as explicit rebuttal of misleading content, can reduce belief in misinformation [5]. However, when added detail elaborates on misinformation, this additive content can increase suggestibility to a greater extent than detail that contradicts the misinformation. For example, Huff and Umanath [6] asked participants to read fictional narratives and answer questions accompanied with additive and contradictory misinformation statements. Questions about the narratives that were accompanied with additive misinformation were endorsed more so than narratives with contradictory misinformation, even with the participants were explicitly advised about and instructed to identify errors in the text. Similarly, Pennycook, Cannon, and Rand [7], incorporating a fake-news paradigm, found that participants still endorsed fake-news articles as accurate even when they had been labelled as contested by fact-checkers. Thus, elaborating on misinformation statements can make them more believable, whilst flagging misinformation as disputed is less effective in reducing believability.

The spread of misinformation is also implicated by individuals’ willingness to share knowledge [8, 9], especially if the validity of the information is not challenged. Pennycook, McPhetres, Zhang, Lu, and Rand [10] found greater sharing of online misinformation about COVID-19 if participants were not questioned as to its accuracy. Vijaykumar et al. [11] also found that COVID-19 statements that were only partially true increased belief in misinformation. Furthermore younger adults showed a greater proclivity to sharing misinformation than older adults, likely due to greater social media use in order to source health information [12] and lower levels of eHealth literacy [13]. In contrast, Greenspan and Loftus [14] found that older adults were more likely to share pandemic-related misinformation than younger adults, as older adults may have more trouble discerning the false information. Older adults more commonly use social media for socializing purposes than sourcing information, which suggests a lesser focus on the accuracy of the content [15]. Thus, age effects regarding sharing misinformation are inconclusive. However, corrective information appears to negate these inconsistencies by increasing intention by both younger and older adults age groups to share factual information about COVID-19 [11].

Building on this evidence, the data we present is a pilot study investigating the following research questions: (1) to what extent does misinformation with narrative elaboration about COVID-19 affect believability in misinformation; (2) to what extent does misinformation with narrative elaboration about COVID-19 affect willingness to share information online; (3) to what extent does the effect of misinformation with narrative elaboration about COVID-19 differ by age. We hypothesized that narrative elaboration would increase believability and willingness to share misinformation about COVID-19, in particular in younger adults.

## Main text

### Method

#### Participants

The sample consisted of 80 participants aged 18 years and over (range 18–74 years, M = 27.71, SD = 12.74), with 63 females, 16 males, and 1 participant who identified as non-binary. There were no other exclusion criteria. Participants were recruited online via social media, SurveySwap, and SurveyCircle. Males and females did not differ significantly in age, t(77) = 0.493, p = 0.624. Participants were also grouped by age (younger = 18–34 years/older = 35 years + ; see [16, 17]). The younger group’s mean age was significantly lower than the older adults’, t(78) = −  23.96, p < 0.001 (see Table 1).

**Table 1:** Mean participant age by gender and by age group

	n	Age range	Mean	*SD*		n	Mean	*SD*
Male	16	19–58	29.19	*14.23*	Younger	66	22.24	*3.01*
Female	63	18–74	27.41	*12.52*	Older	14	53.50	*8.52*
Non-binary	1	23						

#### Materials

An 80-item bespoke questionnaire was developed for this study (see Supplementary Materials 1), containing COVID-19 related statements taken and fact-checked from the Centers for Disease Control and Prevention (https://www.cdc.gov/) [18] and the World Health Organisation (https://www.who.int/) [19] websites. The questionnaire consisted of four conditions: misinformation-only, misinformation with narrative elaboration, truth-only, and truth with factual elaboration. Example items include: *Garlic can cure COVID-19* (misinformation-only): *Garlic can cure COVID-19. Garlic is a healthy food that may have some antimicrobial properties, helping to cure COVID* (misinformation with elaboration); *Antibiotics cannot prevent or treat COVID-19* (truth-only); *Antibiotics cannot prevent or treat COVID-19. COVID-19 is caused by a virus, and therefore antibiotics should not be used for prevention or treatment* (truth with elaboration). Participants read each statement and rated believability using a 1–7 Likert scale where 1 = totally disagree/7 = totally agree, and their willingness to share the information where 1 = no intention at all share/7 = will definitely share (Cronbach’s α = 0.661).

#### Procedure

Ethical approval was granted by the Northumbria University Ethics Committee. Participants accessed the study via Qualtrics (www.qualtrics.com). After providing online consent, participants provided details of age and gender, and then responded to the COVID-19 statements and willingness to share questions.

### Results

Data were analysed using SPSS, v28. Paired samples t-tests were conducted to determine differences in overall believability and willingness to share the statements. Independent samples t-tests were used to compare believability and willingness to share by Group. Correlation analyses investigated relationships between all variables while controlling for age (see Table 2).

**Table 2:** Mean *(SD)* total believability and willingness to share scores by age group

Mean	Misinfo-only	Misinfo Elab	Truth-only	Truth Elab	Misinfo-only Share	Misinfo Elab Share	Truth-only Share	Truth Elab Share
Total	26.66	27.86	45.01	46.71	18.40	18.35	22.73	24.51
*SD*	*7.44*	*8.61*	*10.89*	*11.16*	*10.48*	*10.12*	*13.90*	*15.29*
Younger	27.47	28.82	45.76	47.62	18.32	18.52	22.39	24.12
*SD*	*6.77*	*8.17*	*10.18*	*10.85*	*9.72*	*9.80*	*13.90*	*15.21*
Older	22.86	23.36	41.50	42.43	18.79	17.57	24.29	26.36
*SD*	*9.39*	*9.52*	*13.66*	*12.00*	*13.96*	*11.88*	*14.37*	*16.10*

#### Believability

Believability for misinformation-only statements was significantly lower (M = 26.66, SD = 7.44) compared to misinformation with elaboration (M = 27.86, SD = 8.61), t(79) = − 2.60, *p* = 0.011, *d* = 0.15, and truth-only statements (M = 45.01, SD = 10.89), t(79) = − 12.99, *p* < 0.001, *d* = 1.97. Similarly, believability for misinformation with elaboration (M = 27.86, SD = 8.61) was significantly lower than truth with elaboration (M = 46.71, SD = 11.16), t(79) = − 12.64, *p* < 0.001, *d* = 1.89. Believability for truth-only statements were also significantly lower than truth with elaboration statements, t(79) = − 2.707, *p* = 0.008, *d* = 0.15.

#### Sharing intention

Participants’ willingness to share was significantly lower in response to truth-only (M = 22.73, SD = 13.91) compared with truth with elaboration statements (M = 24.51, SD = 15.29), t(79) = − 3.09, *p* = 0.003, *d* = 0.12. Similarly, willingness to share misinformation-only statements (M = 18.40, SD = 10.48) was significantly lower than truth-only statements (M = 22.73, SD = 13.91), t(79) = − 3.66, *p* < 0.001, *d* = 0.35. Willingness to share misinformation with elaboration (M = 18.35, SD = 10.12) was significantly lower compared to truth with elaboration (M = 24.51, SD = 15.29), t(79) = − 4.49, *p* < 0.001, *d* = 0.46. There was no difference in willingness to share between misinformation-only and misinformation with elaboration statements, t(79) = 0.128, *p* = 0.899, *d* = 0.005.

#### Believability and sharing intention by group

Independent samples t-tests revealed the younger participants gave significantly greater believability ratings compared to older adults to both misinformation-only, t(15.989) = 1.745, p = 0.50, *d* = 0.56, and misinformation with elaboration statements, t(78) = 2.207, p = 0.015, *d* = 0.62. All other analyses by Group were non-significant (p ≥ 0.057).

#### Correlations

In order to explore the relationship between believability, willingness to share, and age, a series of correlation and partial correlation (controlling for age) analyses were carried out (see Table 3). Believability and willingness to share were significantly positively correlated in response to all misinformation (p ≤ 0.013) and truth (p ≤ 0.008) statements, even when controlling for age (misinformation; p ≤ 0.004: truth; p ≤ 0.005). Significant negative correlations were observed between age and believability to misinformation-only (p = 0.007) and misinformation with elaboration (p = 0.004) statements only. All other analyses were non-significant (p ≥ 0.066).

**Table 3:** Partial correlations controlling for age for all variables; correlation of age with all variables

	Misinfo Elab	Misinfo-only Share	Misinfo Elab Share	Truth-only	Truth Elab	Truth-only Share	Truth Elab Share	Age
Misinfo-only	0.866**	0.393**	0.434**	0.038	− 0.018	− 0.031	− 0.023	− 0.299**
Misinfo Elab		0.320**	0.409**	0.131	0.046	− 0.066	0.601**	− 0.315**
Misinfo-only Share			0.946**	− 0.089	− 0.096	0.656**	0.608**	0.081
Misinfo Elab Share				− 0.071	− 0.109	0.665**	0.601**	0.001
Truth-only					0.866**	0.271*	0.269*	− 0.181
Truth Elab						0.234*	0.316**	− 0.206
Truth-only Share							0.941**	0.074
Truth Elab Share								0.066

p < 0.05*; p < 0.01**

#### Summary

Elaboration increased believability of both misinformation and true COVID-19 statements, however participants were equally willing to share misinformation-only and misinformation with narrative elaboration. Younger adults’ believability was significantly greater than older adults to both misinformation-only and misinformation with elaboration. Believability and willingness to share were significantly correlated in all conditions even when controlling for age.

## Discussion

The aim of this pilot study was to investigate believability of and willingness to share misinformation statements about COVID-19 when supported with detailed explanation. Our study expands on the research reporting the effect of additive misinformation in the believability of incorrect information [6]. Here we add to this by demonstrating that adding narrative elaboration to misinformation statements about COVID-19 increases its believability also, especially in younger adults.

Both misinformation and true statements, when supported with detailed elaboration, were more believable than their misinformation-only and truth-only counterparts. Similar patterns were observed by Butterfuss and Kendeou [5] who demonstrated that the addition of factual elaboration to true statements increased believability, as this supplementary detail increased perceptions of its’ accuracy. Here we demonstrated this effect applies to misinformation with narrative elaboration about COVID-19 also. Truth-only and truth with elaboration statements were rated as more believable than both misinformation-only and misinformation with elaboration statements. This is consistent with prior research which also found that truth statements were more believable than misinformation statements in general [5]. However, when considering willingness to share the COVID-19 statements, participants were equally likely to share the misinformation-only and misinformation with elaboration statements. Research has also shown that individuals do not share misinformation with the goal of accuracy, and they are more willing to share when they are not questioned about the accuracy of the statements [10].

Whilst controlling for age did not alter correlations between believability and willingness to share, participant age was negatively correlated with believability for both misinformation-only and misinformation with elaboration statements, and significantly greater in the younger adult age group. This supports existing studies on COVID-19 misinformation and age [11] which also found that younger adults were more likely to believe misinformation than older adults. This is also in line with previous research showing older adults to be less susceptible to misinformation than younger adults [20]. Believability in misinformation is associated with repeated exposure [21] and younger individuals are known to spend more time on social media where COVID-19 misinformation is disseminated frequently [4]. Thus, the findings of the current study adds further understanding regarding the younger age-associated bias towards believability in COVID-19 misinformation. However, the existing literature regarding age and willingness to share misinformation is inconclusive [11, 14], and further emphasised here by the non-significant correlations.

## Limitations

It is possible that the misinformation content in the current study did not align with participants’ existing opinions about COVID-19. Knowledge of COVID-19 is so widespread now that our misinformation statements may not have accurately tapped into misconceptions. Individuals are more likely to endorse misinformation if it is in line with their pre-existing beliefs [14]. Given that much COVID-19 misinformation is spread on social media, prior exposure to both accurate and misinformation was likely high for the participants [7]. A second key limitation was a lack of detail regarding the participants’ conspiracy theory and scientific beliefs (see [10, 21]). Distrust in conventional news outlets increases individuals’ preference to source information from elsewhere such as online media that dissemminates misinformation [22]. Thus understanding of participants’ views regarding information sourcing would help to further elucidate the effect of narrative elaboration on intention to endorse misinformation. Finally, whilst this was a pilot study, the sample size was small. Replication of these findings with a more generalizable sample could have important implications for public health risk communication policy and practice.

## Supplementary Information

**Additional file 1.** Supplementary Materials.

## Data Availability

The data and materials for the study can be accessed via the Northumbria University Figshare service: https://figshare.northumbria.ac.uk/articles/dataset/Narrative_Elaboration_and_misinformation_regarding_COVID-19/19361453/1
