# Supplementary material for: Narrative elaboration makes misinformation and corrective information regarding COVID-19 more believable
Source: BMC Res Notes. 2022 Jun 28;15:235. doi: 10.1186/s13104-022-06134-9 (PMC9241297; doi:10.1186/s13104-022-06134-9)
Supplement: Supplementary file 1 — Additional file 1. Supplementary Materials. [file 13104_2022_6134_MOESM1_ESM.docx]

Stimuli 1

- Misinformation: Garlic can cure COVID-19.
- Misinformation explanation: Garlic can cure COVID-19. Garlic is a healthy food that may have some antimicrobial properties, helping to cure COVID.
- Truth: Garlic cannot cure COVID-19.
- Truth explanation: Garlic cannot cure COVID-19. There is no evidence from the current outbreak that eating garlic has protected people from the new coronavirus.

Stimuli 2

- Misinformation: Antibiotics can prevent or treat COVID-19.
- Misinformation explanation: Antibiotics can prevent or treat COVID-19. Antibiotics have been used to treat cold symptoms, indicating that they would also be effective on COVID-19.
- Truth: Antibiotics cannot prevent or treat COVID-19.
- Truth explanation: Antibiotics cannot prevent or treat COVID-19. COVID-19 is caused by a virus, and therefore antibiotics should not be used for prevention or treatment.

Stimuli 3

- Misinformation: People deciding not to get tested will make it look as though COVID-19 has been wiped out.
- Misinformation explanation: People deciding not to get tested will make it look as though COVID-19 has been wiped out. If people stopped getting tested, the government won’t see the number of cases, and we wouldn’t have more lockdowns.
- Truth: People deciding not to get tested will not make it look as though COVID-19 has been wiped out.
- Truth explanation: People deciding not to get tested will not make it look as though COVID-19 has been wiped out. The number of positive cases isn’t the only measure used to understand the spread of the virus. The number of tests and things like test positivity are also used.

Stimuli 4

- Misinformation: Reopening universities will cause 50,000 deaths
- Misinformation explanation: Reopening universities will cause 50,000 deaths. This could be caused without strong controls to prevent the spread of COVID-19.
- Truth: Reopening universities will almost certainly not cause 50,000 deaths.
- Truth explanation: Reopening universities will almost certainly not cause 50,000 deaths. This comes from a research paper that has not been peer-reviewed. It is based on several assumptions, including that every student gets infected, and nothing is done to stop it.

Stimuli 5

- Misinformation: A Danish study on masks states they have no significant effect.
- Misinformation explanation: A Danish study on masks states they have no significant effect. The study found the COVID-19 infection rate was not significantly different between mask-wearers and non-mask wearers.
- Truth: A Danish study on masks only tells half the story on masks having no significant effect.
- Truth explanation: A Danish study on masks only tells half the story on masks having no significant effect. This study shows no significant difference between mask-wearers and non-mask wearers. However, this is not proof that masks don’t have a significant effect, as the study didn’t look at whether mask-wearing protects others.

Stimuli 6

- Misinformation: Aluminum in vaccines can cause Alzheimer’s disease.
- Misinformation explanation: Aluminum in vaccines can cause Alzheimer’s disease. Aluminum has been known to cause Alzheimer’s disease.
- Truth: Aluminum in vaccines cannot cause Alzheimer’s disease.
- Truth explanation: Aluminum in vaccines cannot cause Alzheimer’s disease. There is no link between Alzheimer’s disease and the small amount of aluminum found in some vaccines has been established.

Stimuli 7

- Misinformation: Contact tracing has stopped in schools.
- Misinformation explanation: Contact tracing has stopped in schools. Contact tracers have been told not to trace contacts of COVID-19 cases connected to educational settings.
- Truth: Contact tracing has not stopped in schools.
- Truth explanation: Contact tracing has not stopped in schools. This is incorrect. Contact tracers have been told to not escalate these cases to local health teams, but their contacts will still be traced nationally.

Stimuli 8

- Misinformation: The government is planning to roll out an untested COVID-19 vaccine.
- Misinformation explanation: The government is planning to roll out an untested COVID-19 vaccine. The government is bringing in a law that means unlicensed, untested vaccines can be given to people and no one is liable if they go wrong.
- Truth: The government is not planning to roll out an untested COVID-19 vaccine
- Truth explanation: The government is not planning to roll out an untested COVID-19 vaccine. The government was holding a consultation on whether it should change rules around unlicensed vaccines. Any vaccine given to the public will have to go through several stages of testing, regardless of this consultation.

Stimuli 9

- Misinformation: Ticketmaster will demand proof of a COVID-19 vaccine for concert entry.
- Misinformation explanation: Ticketmaster will demand proof of a COVID-19 vaccine for concert entry. Ticketmaster is planning a mandatory COVID-19 verification policy for concert attendance.
- Truth: Ticketmaster won’t demand proof of a COVID-19 vaccine for concert entry.
- Truth explanation: Ticketmaster won’t demand proof of a COVID-19 vaccine for concert entry. Ticketmaster said it is exploring a number of safety features that event organisers may want to use, including checks on test and vaccine status, but entry requirements will be up to organisers.

Stimuli 10

- Misinformation: The number of people dying right now is the same as in any other year.
- Misinformation explanation: The number of people dying right now is the same as in any other year. About 1% of the population is infected with COVID-19 today.
- Truth: The number of people dying right now is not the same as in any other year.
- Truth explanation: The number of people dying right now is not the same as in any other year. According to the most recent data, about 1% of the population having COVID-19 is correct. However, the average amount of people dying is higher than in an average year (14.3% higher in England that the 5-year average).
